# Effect of acid proticity on the thermodynamic parameters of charge transfer resistance in corrosion and passivation of nickel based glass alloy

**DOI:** 10.1038/s41598-024-52036-0

**Published:** 2024-01-20

**Authors:** Khadijah M. Emran, Noureddine Ouerfelli

**Affiliations:** 1 Chemistry Department, college of Science, Taibah University, Medina, Saudi Arabia; 2 Institut Supérieur des Technologies Médicales de Tunis, LR13SE07, Laboratoire de Biophysique et Technologies Médicales, Université de Tunis El Manar, Tunis, Tunisia

**Keywords:** Chemistry, Engineering, Materials science

## Abstract

The effect of temperature on electrochemical properties of Ni_82.3_Cr_7_Fe_3_Si_4.5_B_3.2_ glassy alloy in different acid proticity has been investigated utilizing AC and DC methods. Firstly, the handling of experimental data on the temperature dependence of charge transfer resistance, as well as corrosion current density permits us to determine the values of classical Arrhenius parameters as well as the thermodynamic ones considered approximately independent of temperature. This leads us to deduce a global interpretation on the phenomenon of corrosion and polarization. Secondly, the deviation to the linearity of the Arrhenius behavior and the real dependence on temperature of the thermodynamic parameters, permit us to clearly quantify the effect of the acid proticity and define, for the first time, the concept of current Arrhenius parameters and the current thermodynamic ones, as well as the modeling of the enthalpy–enthalpy compensation. Moreover, the effect of temperature can be investigated using the Vogel–Fulcher–Tammann model to reveal that the corresponding Vogel temperature has an interesting physical meaning.

## Introduction

The high corrosion resistance features that often extend an improved corrosion resistance of metallic glasses which chemically afford a smooth contacting metallic surface with minimal defects and free of voids, dislocations and/or atomic terraces, so they have no crystallographic dislocations, crystal imperfections, distortions, grain boundaries or secondary phase elements^1–3^. Passivation is the process of making a material “non-reactive” in relation to another material^4,5^. After corrosion process, passivation is the spontaneous formation of a hard non-reactive surface film that inhibits the corrosion process.

High temperature corrosion plays an increasingly important role in the selection of materials, in particular within the power generation industry. The most influencing factors on susceptibility to corrosion of metallic glasses are the nature of the components and high temperatures, either in acidic or alkaline media^6–10^.

For nickel-contained glassy alloys, the effects of microstructure change on the corrosion behaviors of Ni_55_Nb_20_Ti_10_Zr_8_Co_7_ glassy alloy were investigated in 1 mol/L HCl and 0.5 mol/L H_2_SO_4_ solutions^11^. Anodic polarizations, at range 872 K to 1050 K, reveal the high corrosion resistance for all the alloys, which can be due to the formation of a passive film on the alloy surface corrosion behavior of Al_86_Ni_10_Y_4_ and Al_83_Ni_13_Y_4_ amorphous alloys which was investigated in 3.5 *wt*.% NaCl solution^12^. The electrochemical techniques reveal the rapidly solidified Al_86_Ni_10_Y_4_ and Al_83_Ni_13_Y_4_ alloys which exhibit better passivity and higher polarization resistance after annealing at 150 °C.

The effects of Mo and Cr substitution for Ni on Ni_77−*x*−*y*_Mo_*x*_Cr_*y*_Nb_3_P_14_B_6_ (*x* = 5–9, *y* = 0–5) glassy alloy corrosion have been studied in 1 M NaCl and 1 M HCl solutions. The addition of the appropriate Mo and Cr content as much as possible are beneficial for the enhancement of the corrosion resistance of the present Ni-based (bulk metallic glasses) BMGs with an *I*_*corr*_ in the order of 10^−6^ A/cm^2^ and a *R*_*corr*_ about 10^−3^ mm/year in both solutions^13^.

In a recent research, Han et al.^14^ studied the high temperature oxidation behaviors for Ir_35_Ni_25_Ta_40_ and Ir_35_Ni_20_Ta_40_B_5_ (*wt*%) metallic glasses that become the optimal candidates for the molding materials of optical devices. It was found that Ir–Ni–Ta-(B) MGs display good oxidation resistance and there appears a unique four-layer oxide micro-structure on the surface and the natural oxide Ta_2_O_5_ will be transformed into TaO_2_ with the gradual increase of temperature after approaching *T*_g_ of Ir–Ni–Ta-(B) MGs^14^. In another study, the corrosion behavior of Ni_62_Nb_33_Zr_5_ bulk metallic glasses (BMGs) after annealing treatment (AT) at different crystallization temperatures and cryogenic treatment (CT) at –100 °C are experimentally investigated^15^. Superior corrosion resistance is obtained in the cryo-treated BMG because of the high degree of amorphization. The passive film is found to be composed mainly of Nb_2_O_5_ and ZrO_2_, demonstrating that Nb and Zr are conducive to reacting with oxygen to form a passive film. Globally, several recent works treated different conductive and corrosion behaviors in some BMGs materials^16–21^.

In our previous study, a systematic study of the corrosion and passivation behavior of the Ni_82.3_Cr_7_Fe_3_Si_4.5_B_3.2_ (*wt*%) in 3.0 mol/L aqueous solutions of HCl, H_2_SO_4_, and H_3_PO_4_ acids solutions at temperatures range (20–80) was carried. The passive film on Ni_82.3_Cr_7_Fe_3_Si_4.5_B_3.2_ (*wt*%) glassy alloy surfaces was a uniform and stable chromium oxy-hydroxide [CrO_*x*_(OH)_3−2*x*_·nH_2_O], which is a few atoms thick^1^. In recent research^22^ we have extended the Arrhenius-type expression by one term in 1/*T*^2^ and collected some physical meaning to the new related coefficients for which it is found that they depend closely on the number of acid hydrogen atoms in the polyacid for the corrosion and passivation of the nickel based metallic glass alloy of the composition Ni_82.3_Cr_7_Fe_3_Si_4.5_B_3.2_. We have suggested a mathematical formula that allows indirect calculation of the familiar Arrhenius activation energy using only the parameters of the homographic model for each acid separately or for all three polyacids together.

As a continuation of our previous works^1,22^ and, based on the above considerations, we suggest some novel empirical models for thermodynamic parameters in the present work. Therefore, we will introduce a new concept of current quantities after considering the dependence with temperature of the Arrhenius and thermodynamic parameters. Moreover, the novel application of the Vogel–Fulcher–Tammann (VTF) model^14,23–28^ on corrosion reveals new physical meanings of the corresponding parameters. Also, the effect of the proton numbers, of the used polyacids, on the thermodynamic parameters is modeled with logarithm form.

## Materials and methods

Ni-based alloy ingots Ni_82.3_Cr_7_Fe_3_Si_4.5_B_3.2_ (*wt*%) alloy was prepared by rapid solidification supplied as ribbons of about 2.5–7.5 mm length and 20–50 µm thickness by Vacuumschmelze after polishing operation.

The electrochemical cell included three electrodes such as: the corroded sample as working electrode, a platinum-wire counter electrode and, a saturated calomel reference electrode for which each experiment is realized using a new alloy strip after degreasing in alcohol, rinsing with be-distilled water, and ultrasonic cleaning.

Because of the high aggressivity of acids in the studied temperature range, electrochemical characterization was performed in acidic solutions of 3.0 mol/L of HCl, H_2_SO_4_, or H_3_PO_4_ for examining the electrochemical behavior of Ni-based glassy alloy at different temperatures ranging from 20 to 80° before the specimens were destroyed notably at higher temperature.

The electrochemical measurements, on samples with 2 cm^2^ surface area, were performed by direct and alternating currents at several temperatures with an ACM Gill AC instrument. Measurements by electrochemical impedance spectroscopy were carried out at frequencies from 0.1 Hz to 30 kHz utilizing sinusoidal wave of 5 mV amplitude. Polarization measurements were realized, at a scan rate of 2 mV/s, from (− 800 to 2000) mV.

Complementary details are presented in our previous works^1,22^.

## Deviation to Arrhenius behavior

### Correlation between Arrhenius parameters

Activation energy control occurs when the electrode kinetics or corrosion rate is controlled by a slow electrochemical step. The activation energy (*Ea*) can be determined from Arrhenius plot that often used to analyze the effect of temperature on the rates of chemical reactions where the reactants molecules colliding probability becomes higher and the reaction proceeds faster at higher temperatures. In our study *Ea* of the corrosion process of glassy alloy in 3.0 mol/L solutions of the studied acids was obtained from the assumed linear variation of the corrosion reaction rate assigned as the reciprocal of the charge transfer resistance, 1/*R*_*ct*_, with temperature^1,22^.

The variation of the charge transfer resistance (*R*_ct_) as a function of temperature (*T*) is considered following the Arrhenius-type equation (Eqs. 1 or 2). In this context, in previous works^1,22^ we have applied the Arrhenius-type equation in this exponential and logarithm form where the two Arrhenius parameters are generally supposed both constants practically independent of temperature.

1 $${R}_{ct}={A}_{ct}{e}^{\frac{{E}_{a}}{RT}}$$

2 $${\text{ln}}{R}_{ct}={\text{ln}}{A}_{ct}+\frac{{E}_{a}}{R}\times \frac{1}{T}$$

where *Ea* is the activation energy, *A*_ct_ is the pre-exponential factor (Table 1). Though the theories of collision, transition state, statistical physics, theory or chemical reaction rate have detailed and expressed the effect of temperature, generally^1,22^, experimenters assumed that these two Arrhenius parameters are both constants and practically independent of temperature, where the plot of (ln*R*_ct_) as a function of the reciprocal of absolute temperature (1/*T*) gives approximately a straight line^1,22^ whose the slope is equal to (*Ea*/*R*) and the intercept on the ordinate is equal to (ln*A*_ct_). Additionally, we can append that in addition to the *y*-intercept (ln*A*_ct_) in (Fig. 1 of Ref.^22^), we can also speak of the *x*-intercept (*T*_*A*_ = –*Ea*/(*R*·ln*A*ct)) previously named as the Arrhenius temperature^22^. In addition, we have shown that (− *R*·ln*A*_ct_) is considered as an entropic factor (Fig. 3 of Ref.^22^) and closely correlated with the activation entropy Δ*S* determined from impedance measurements in our earlier work^1^ (Table S1).

**Table 1:** Arrhenius parameters (*Ea* and ln*A*_ct_), the entropic factor of Arrhenius—*R*·ln(*A*_ct_) from linear regression of Eq. 2, the activation entropy Δ*S* and Arrhenius temperature (*T*_*A*_).

Acid	*E*_a_/kJ mol^−1^^1^	ln(*A*_ct_/Ω cm^2^)^1^	– *R*·ln*A*_ct_/J.K^−1^ mol^−1^^22^	Δ*S*/J K^−1^ mol^−1^^1^*	*T*_*A*_/K^22^
HCl	52.976	− 12.310	102.35	− 151.0	517.59
H_2_SO_4_	83.287	− 24.546	204.09	− 52.0	408.10
H_3_PO_4_	102.27	− 31.733	263.84	19.0	387.61

*Calculated via the variation of the charge transfer resistance (*R*_ct_/Ω cm^2^) as a function of temperature (*T*).

**Figure 1:**
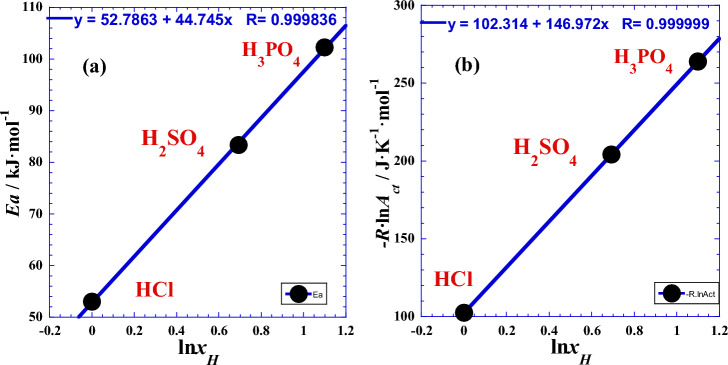
Variation against the logarithm of the acid proticity (ln*x*_H_) of (**a**): the activation energy (*Ea*) and (**b**): the entropic factor of Arrhenius—*R*·ln(*A*_ct_).

By similarity of some recent studies on the viscosity of liquid state which find that the Arrhenius temperature (*T*_*A*_) is strongly correlated to the boiling temperature (*T*_*b*_)^29–31^, and regarding that the BMGs are in solid state, we can assume that the Arrhenius temperature (Table 1) is probably in causal correlation with the corresponding glass transition temperature (*T*_*g*_) which is strongly correlated with the melting point (*T*_*m*_)^14,26,27^. This allows us in the future to make certain predictions, estimations and comparisons of different BMGs features.

Furthermore, the mutual correlation between the two Arrhenius parameters (*Ea* and ln*A*_ct_) (Table 1), shows quasi-linearity inter-dependence expressed as follows:

3 $${E}_{a}={E}_{a0}+{\tau }_{0}\cdot (- R{\text{ln}}{A}_{ct})$$

where (*E*_a0_ = 21.64 kJ mol^−1^) is the activation energy corresponding the zero value of the entropic factor, and the slope (*τ*_0_ = 304.45 K) is a temperature characteristic of the studied system at such conditions. Numerical applications require the use of the following convenient simple formulas:

$$\begin{aligned} E_{{\text{a}}} ({\text{kJ}}\cdot{\text{mol}}^{ - 1} ) \, = & \, 21.64 \, {-} \, 2.53134 \times {\text{ln}}\left( {A_{{{\text{ct}}}} /\Omega \cdot{\text{cm}}^{2} } \right) \\ {\text{ln}}\left( {A_{{{\text{ct}}}} /\Omega \cdot{\text{cm}}^{2} } \right) \, = & \, 8.5488 \, {-} \, 0.39505 \times E_{{\text{a}}} ({\text{kJ}}\cdot{\text{mol}}^{ - 1} ) \\ \end{aligned}$$

We point out that the values of coefficients can vary according to experimental conditions and to the system characteristics and specificity.

We notice that in the general case when the two Arrhenius parameters (*Ea* and ln*A*_ct_) depend slightly on the temperature, the characteristic temperature (*τ*_0_) becomes the derivative of the activation energy with respect of the entropic factor (Eq. 4) at constant pressure.

4 $${\tau }_{0} = -\frac{1}{R}{\left(\frac{\partial Ea}{\partial ({\text{ln}}{R}_{ct})}\right)}_{P}$$

Since the observed linear dependence between the two Arrhenius parameters *Ea* and ln*A*_ct_ (Eq. 3), the charge transfer resistance *R*_ct_ can be expressed only against of *Ea* or ln*A*_ct_ (Eqs. 5 and 6), respectively. Indeed, combining (Eqs. 2 and 3) we can write the following expressions:

5 $${\text{ln}}{R}_{ct}=\frac{{E}_{a0}}{R{\tau }_{0}}+\frac{{{\varvec{E}}}_{{\varvec{a}}}}{R}(\frac{1}{T}-\frac{1}{{\tau }_{0}})$$

Or,

6 $${\text{ln}}{R}_{ct}=\frac{{E}_{a0}}{RT}+\frac{T-{\tau }_{0}}{T}\cdot \mathbf{l}\mathbf{n}{{\varvec{A}}}_{{\varvec{c}}{\varvec{t}}}$$

Numerical applications require the use of the following convenient simple formulas:

$${\text{ln}}{(R}_{ct}/\Omega {{\text{cm}}}^{2})=8.5488+\frac{{({\varvec{E}}}_{{\varvec{a}}}/\mathrm{kJ }{{\text{mol}}}^{-1})}{R/1000}(\frac{1}{T}-\frac{1}{304.45})$$

$${\text{ln}}{(R}_{ct}/\Omega {{\text{cm}}}^{2})=\frac{2602.7}{(T/{\text{K}})}+\frac{T-304.45}{T}\cdot \mathbf{l}\mathbf{n}{({\varvec{A}}}_{{\varvec{c}}{\varvec{t}}}/\Omega {{\text{cm}}}^{2})$$

The values of these coefficients can vary according to experimental conditions and to the characteristics and specificity of the studied system. In addition these two expressions are interesting when only one Arrhenius parameter is predicted by certain theory, the charge transfer resistance *R*_ct_ can be then estimated by (Eqs. 5 or 6).

To our knowledge, there is no theoretical and physical basis of this observed causal correlation or any developed estimative techniques for our original suppositions. Then, we will be able to give more closely our checking after applying these suggested equations by several researchers in the future.

Based on the studied correlation^26,27^ between the glass transition temperature (*T*_*g*_) and the melting temperature (*T*_*m*(*AxBy*)_) of the base components of various metallic glasses where (300 K < *T*_*g*_ < 900 K) and (600 K < *T*_*m*(*AxBy*)_ < 1600 K) and the suggested expressions for the viscosity-temperature dependence^29–31^, we propose similar predictive expression form for the charge transfer resistance–temperature dependence expressed as follows.

7 $$\frac{1}{{T}_{A}}-\frac{1}{{T}_{m}}\approx \frac{1}{{T}_{g}}$$

As quick application of this formula, if we take (*T*_*m*_ = 1726 K) for some Ni-MGs in (Fig. 1 of Ref.^26^) and the Arrhenius temperature (*T*_*A*_ = 517.59 K) in the monoacid medium for our Ni-MG (Table 1), we find, via the (Eq. 7), approximately a value of (*T*_*g*_ = 739 K) which is included in the range of the studied set of Ni-MGs in (Fig. 1 of Ref.^26^). We conclude that this approximative estimation can open an interesting path for future investigators to test, valid and improve the proposed model (Eq. 7).

In the same way, by analogy with the viscosity-temperature dependence at liquid phase^29–31^ which authors have discovered that the *A*_ct_ is the pre-exponential factor (Eq. 1) is very close to the viscosity of the same system at vapor phase and at normal boiling temperature, we can presume that our pre-exponential factor (*A*_ct_) in (Table 1) probably represents approximately the charge transfer resistance (*R*_ct_) in liquid phase at the melting temperature (*T*_*m*_) or at the glass transition temperature (*T*_*g*_) under atmospheric pressure.

### Effect of protons’ number (***x***_H_)

For this query, and to test the effect of number of protons (*x*_H_) of the acids (H_*x*_B), we plotted the two Arrhenius parameters against the logarithm of the acid proticity (ln*x*_H_). Figure 1 shows a spectacular linearity, which leads us to wonder, for future investigations, if this relationship is valid for other strong acids with the same proticity. The slope of the straight line in Fig. 1a represents the activation energy gap *ε*_*g*_ (Table 2) and it corresponds to the jump of energy value when the proticity increases with unity. The same ascertainment is valid for the Fig. 1b concerning the entropic factor gap *σ*_*g*_ (Table 2). This observed linearity can be expressed as follows:

8 $${E}_{a}({x}_{H})={E}_{a1}+{\varepsilon }_{g}\cdot {\text{ln}}{x}_{H}$$

9 $$- R{\text{ln}}{A}_{ct}({x}_{H})=- R{\text{ln}}{A}_{ct1}+{\sigma }_{g}\cdot {\text{ln}}{x}_{H}$$

where the straight line parameters are the intercepts on the ordinate (*E*_a1_) and (– *R*·ln*A*_ct1_) correspond to the values of the activation energy and entropic factor related to the monoacid HCl (*x*_H_ = 1), respectively (Table 2). Numerical applications require the use of the following convenient simple formulas:

**Table 2:** Straight lines parameters from linear regression of Eqs. 8 and 9.

*ε*_*g*_	*E*_a1_	*σ*_*g*_	– *R*·ln*A*_ct1_
kJ mol^−1^	kJ mol^−1^	J.K^−1^ mol^−1^	J K^−1^ mol^−1^
44.745	52.786	146.972	102.314
	52.976*		102.35*

*Correspond to HCl experimental data^1^, see Table 1.

$${E}_{a}({x}_{H})/\mathrm{kJ}\,{{\text{mol}}}^{-1}=52.976+44.745\cdot {\text{ln}}{x}_{H}$$

$${\text{ln}}{(A}_{ct}\left({x}_{H}\right)/\Omega\, {{\text{cm}}}^{2})=-12.310-17.677\cdot {\text{ln}}{x}_{H}$$

The values of these coefficients can vary according to the experimental conditions as well as the system characteristics and specificity.

However, Eqs. 1, 8 and 9 can be re-expressed to explicit the effect of protons number (*x*_H_) of the polyacids (H_*x*_B) based on monoacid HCl (*x*_H_ = 1) data.

10 $${A}_{ct}({x}_{H})={A}_{ct1}{{\cdot x}_{H}}^{\frac{-{\sigma }_{g}}{R}}$$

11 $${R}_{ct}({x}_{H})={R}_{ct1}{{(T)\cdot x}_{H}}^{\frac{{\gamma }_{g}}{RT}}$$

12 $${\gamma }_{g}={\varepsilon }_{g}-T\cdot {\sigma }_{g}$$

where *γ*_*g*_ represents a kind of free energy gap of acid protonation, *R*_ct1_(*T*) is the charge transfer resistance related to the monoacid HCl (*x*_H_ = 1) at given temperature (*T*) and (*A*_ct1_) is the corresponding pre-exponential factor (Eq. 1).

Numerical applications require the use of the following convenient simple formulae:

$${(A}_{ct}\left({x}_{H}\right)/\Omega {{\text{cm}}}^{2})=4.5266\times {10}^{-6}{{\cdot x}_{H}}^{-17.677}$$

$${R}_{ct1}(T)/\Omega {{\text{cm}}}^{2}=4.5266\times {10}^{-6}{e}^{\frac{6348.7}{(T/{\text{K}})}}$$

$${\gamma }_{g}/\mathrm{kJ }\,{{\text{mol}}}^{-1}=44.745-0.14697\cdot (T/{\text{K}})$$

$${R}_{ct}({x}_{H})/\Omega {{\text{cm}}}^{2}=4.5266\times {10}^{-6}{e}^{\frac{6348.7}{T}}{{\cdot x}_{H}}^{\frac{{\gamma }_{g}}{RT}}$$

The values of these coefficients can vary according to the experimental conditions as well as the system characteristics and specificity (Table S2).

We expect that these suggested expressions can be utilized in future estimations for other experimental conditions or other studied materials. We also conclude that the proticity (*x*_H_) of the polyacid (H_*x*_B) has a substantial effect, which can be modeled for future prediction, or estimation and can induce theorists to develop or improve theories^1,16–22,32–37^.

**Figure 2:**
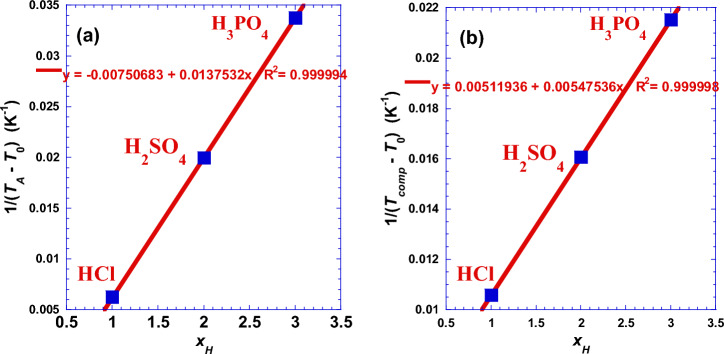
Variation against the acid proticity (*x*_H_) correlation between (**a**): the Arrhenius temperature (*T*_*A*_) and (**b**): the temperature of compensation (*T*_*comp*_).

Moreover, using linearization technique, we detect another interesting strong correlation between the Arrhenius temperature (*T*_*A*_) and the compensation temperature (*T*_*comp*_) with the acid proticity (*x*_H_) depicted in Fig. 2.

Linearization technique leads to simple numerical applications requiring the use of the following convenient simple formulas:

$$\frac{1}{{T}_{A}- 358}\approx \frac{{x}_{H}}{72.71}-\frac{1}{133.21}$$

and

$$\frac{1}{{T}_{comp}- 223}\approx \frac{{x}_{H}}{182.64}-\frac{1}{195.34}$$

where

*T*_*comp*_ =|Δ*H°*/Δ*S°*|

And the six numerical values are all equivalent to absolute temperatures expressed in Kelvin. We hope that in future works we will find some further interpretations for probable physical significance of the amount of these values, especially for (*T*_0_) indicated in the first member of the two previous equations (Table S3).

### Activation energy-temperature dependence

Nevertheless, for the variation of the logarithm of charge transfer resistance (ln*R*_ct_) as a function of the inverse of the reciprocal temperature (1/*T*), we have observed feeble net deviations from the linearity of the Arrhenius behavior in some results in literature^1,22,33^. Generally, the linear regression used by the majority of experimenters is just an approximation, because the experimental points are not aligned in practically all researches. For this reason, we consider the slight variation of activation energy with the temperature around the constant values calculated by the classical Arrhenius-type equation, and then the interpretation of the sense of variation against temperature will be very interesting and enrich classical conclusions, discussions and explanations. To differentiate between the classical Arrhenius parameters independent of temperature and those of the new concept, we will name them as current Arrhenius parameters *E*a(*T*) and ln*A*_ct_(*T*) which are dependent on temperature. So, Eq. 1 is dropped and replaced by a similar expression whose parameters become temperature dependent (Eq. 13).

13 $${\text{ln}}{R}_{ct}={\text{ln}}{A}_{ct}(T)+\frac{{E}_{a}(T)}{R}(\frac{1}{T})$$

For that, we propose, as optimization by nonlinear regression, to simply fit the experimental results (ln*R*_ct_) with respect of (1/*T*) with only a small-degree polynomial, which can be expressed in its general form as follows:

14 $${\text{ln}}{R}_{ct}={a}_{0}+{a}_{1}\left(\frac{1}{T}\right)+{a}_{2}{\left(\frac{1}{T}\right)}^{2}+{a}_{3}{\left(\frac{1}{T}\right)}^{3}+\dots +{a}_{n}{\left(\frac{1}{T}\right)}^{n}$$

In fact, in our situation, we are satisfied with two-degree polynomial (*a*_3_ = 0, etc.) where results are given in Table 3^22^.

**Table 3:** Optimal adjustable coefficients (*a*_*i*_) from nonlinear regression of Eq. 14.

Acid	*a*_0_	*a*_1_	*a*_2_	*R*-square	*χ*-square
	–	K	K^2^	–	–
HCl	38.2266	− 2.61907 × 10^4^	5.22150 × 10^6^	0.99929	0.00073
H_2_SO_4_	19.7075	− 1.84969 × 10^4^	4.57233 × 10^6^	0.99987	0.00017
H_3_PO_4_	− 2.1065	− 6.78931 × 10^3^	3.06105 × 10^6^	0.99753	0.00153

We note that, the mathematical derivation of Eq. 14 can lead us to determine (*E*a) and (ln*R*_ct_) using the following equations,

15 $${E}_{a}(T) = R{\left(\frac{\partial ({\text{ln}}{R}_{ct})}{\partial (1/T)}\right)}_{P}$$

16 $${\text{ln}}{A}_{ct}\left(T\right)= {\left(\frac{\partial \left(T\cdot {\text{ln}}{R}_{ct}\right)}{\partial \left(T\right)}\right)}_{P}={\text{ln}}{R}_{ct}-\frac{{E}_{a}(T)}{RT}$$

17 $${E}_{a}\left(T\right)={[a}_{1}+2{a}_{2}\left(\frac{1}{T}\right)+3{a}_{3}{\left(\frac{1}{T}\right)}^{2}+ \dots +n{a}_{n}{\left(\frac{1}{T}\right)}^{n-1}]R$$

18 $${\text{ln}}{A}_{ct}\left(T\right)={a}_{0}+{2a}_{1}\left(\frac{1}{T}\right)+{3a}_{2}{\left(\frac{1}{T}\right)}^{2}+{4a}_{3}{\left(\frac{1}{T}\right)}^{3}+\dots +(n{+1)a}_{n}{\left(\frac{1}{T}\right)}^{n}$$

In our case, the deviation to the linearity is feeble, so we will consider only a second degree polynomial in our nonlinear regression and consider with excellent approximation that the third coefficient is zero (*a*_3_ = 0)^1^. Numerical applications require the use of the following convenient simple formulas:

$${E}_{a}\left(T\right)/k\mathrm{J }{{\text{mol}}}^{-1}={[a}_{1}+2{a}_{2}\left(\frac{1}{T}\right)]R/1000$$

$${\text{ln}}{(A}_{ct}\left(T\right)/\Omega {{\text{cm}}}^{2})={a}_{0}+{2a}_{1}\left(\frac{1}{T}\right)+{3a}_{2}{\left(\frac{1}{T}\right)}^{2}$$

where we can inject, for each acid, the values of (*a*_*i*_) from Table 3. The values of these coefficients can vary according to the experimental conditions as well as the system characteristics and specificity.

**Figure 3:**
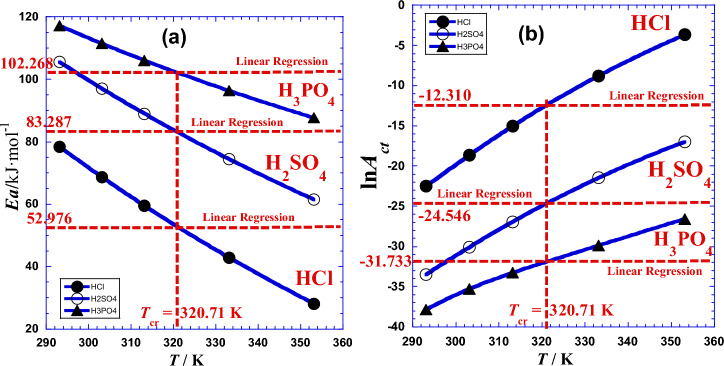
Variation as a function of temperature (*T*) of, (**a**): the current activation energy *Ea* (kJ/mol) and (**b**): the logarithm of the current pre-exponential factor (ln*A*_ct_) for different acids. (●): HCl; (○): H_2_SO_4_; (▲): H_3_PO_4_.

Results of nonlinear regression are given in Table 4. Figure 3 illustrates this interesting variation of Arrhenius parameters with temperature. We add that we will be forced to augment the polynomial-degree when the general trend of data points, in the plot of ln*R*_*ct*_ = *f*(1/*T*) has a strong curvature or a change of curvature (inflection point).

**Table 4:** Results of values of the two current Arrhenius parameters *E*a(*T*) and ln*A*_ct_(*T*) obtained by Eqs. 17 and 18.

***T***	***T***	ln*R*_ct_	Linear Regression		Non Linear Regression	
			*E*a	ln*A*_ct_	*E*a(*T*)	ln*A*_ct_(*T*)
°C	K	–	kJ mol^−1^	–	kJ mol^−1^	–
HCl						
20	293.15	9.6747	52.976	− 12.310	78.428	− 22.502
30	303.15	8.6028			68.658	− 18.637
40	313.15	7.8220			59.511	− 15.034
47.56^a^	320.71^a^	7.3389^c^			52.85^b^	− 12.355^b^
60	333.15	6.7072			42.866	− 8.7680
80	353.15	5.9092			28.105	− 3.6625
			*R*^2^ = 0.98176^d^		*σ* = 0.0260^e^	*σ* = 0.0057^e^
H_2_SO_4_						
20	293.15	9.8341	83.287	− 24.546	105.57	− 33.480
30	303.15	8.4154			97.018	− 30.076
40	313.15	7.2657			89.009	− 26.920
47.56^a^	320.71^a^	6.5191^c^			83.34^b^	− 24.569^b^
60	333.15	5.4067			74.433	− 21.465
80	353.15	3.9823			61.508	− 16.965
			*R*^2^ = 0.99440^d^		*σ* = 0.0279^e^	*σ* = 0.0127^e^
H_3_PO_4_						
20	293.15	10.244	102.268	− 31.733	117.19	− 37.836
30	303.15	8.9660			111.46	− 35.255
40	313.15	7.4844			106.10	− 33.265
47.56^a^	320.71^a^	6.5063^c^			102.24^b^	− 31.798^b^
60	333.15	4.9097			96.341	− 29.871
80	353.15	3.2910			87.688	− 26.573
			*R*^2^ = 0.99589^d^		*σ* = 0.0389^e^	*σ* = 0.0173^e^

(a): Crossover temperature *T*_*cr*_ (Fig. 3). (b); current Arrhenius parameters *E*a(*T*) and ln*A*_ct_(*T*) calculated with Eqs. 17 and 18, respectively. (c): logarithm of charge transfer resistance (ln*R*_ct_) estimated with Eq. 13. (d): from^22^. (e): Standard deviation *σ.*

Graphical analysis of (Fig. 3) shows that values of the current Arrhenius parameters *E*a(*T*) and ln*A*_ct_(*T*) meet those of classical Arrhenius parameters *E*a and ln*A*_ct_ (Table 4) for practically a unique temperature (*T*_*cr*_ = 320.71K, 47.56 °C) named as crossover temperature which is approximately equal to the arithmetic mean (319.15K, 46 °C) of the set of five working temperatures indicated in (Table 4). We can notice that this temperature can be an excellent working temperature for our studied system because it gives precise values of Arrhenius parameters whether we treat the data with linear or non-linear regression.

We add that the crossover temperature (*T*_*cr*_) is not specific for the system like the characteristic temperature (*τ*_0_) studied in previous work^1^, one must be careful because it cannot be characteristic of the studied system; it is only an intermediate mathematical variable obeying Eq. 19 by simply indicating approximately the temperature of the system during its process. The Eq. 19 can be easily obtained by equalizing Eq. 1 for classical parameters and Eq. 17 for current ones.

19 $${T}_{cr}= \frac{2{a}_{2}R}{{E}_{a}-{a}_{1}R}$$

where (*Ea*) represents the activation energy obtained by linear regression. For numerical applications, we can inject, for each acid, the values of (*a*_*i*_) from Table 3 and those of (*Ea*) obtained by linear regression from Table 4. Moreover, we can also use the values of energy parameters determined in our previous work^1^ as follows,

20 $${T}_{cr}= \frac{2{{E}_{2}}^{2}}{({E}_{a}+{E}_{1})R}$$

We notice that the middle-temperature (*T*_*md*_ = 323.15K, 50 °C) of the studied temperature range is very close to the temperature parameter (*T*_*cr*_) as it is shown in Table 4. Indeed, to give an approximate estimation of the mean activation energy *Ea* that should be obtained by classical linear regression we can apply the following reasoning. We can calculate the average value of the function *Ea*(*T*) expressed by Eq. 17 over a temperature domain [*T*_min_,*T*_max_] like in our situation [293.15,353.15]K, by the following expression:

21 $$\overline{{E}_{a}}=\frac{1}{{T}_{max}-{T}_{min}}{\int }_{{T}_{min}}^{{T}_{max}}{E}_{a}(T)dT$$

Which can be adapted for Eq. 21 and can lead to a convenient expression (Eq. 22) for an average value of activation energy without using direct linear regression of ln*R*_ct_ with 1/*T*.

22 $$\overline{{E}_{a}}=R[{a}_{1}+2{a}_{2}\frac{ln\left(\frac{{T}_{max}}{{T}_{min}}\right)}{{T}_{max}-{T}_{min}}]$$

**Figure 4:**
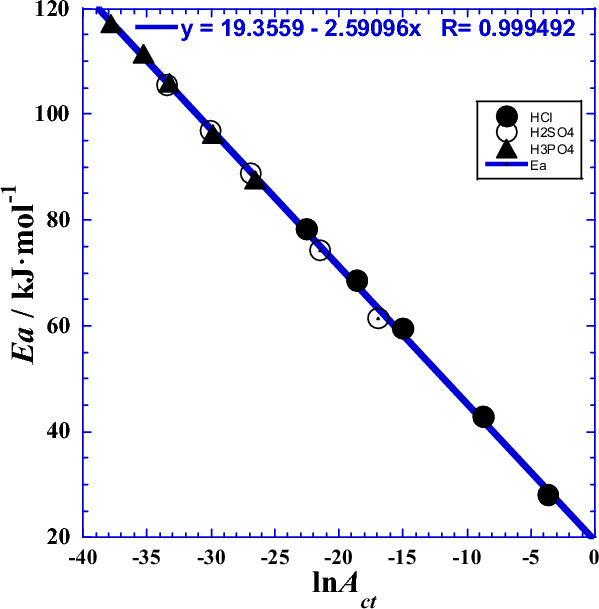
Correlation between the current activation energy *Ea* (kJ/mol) from polarization and impedance measurements^1^ and the current entropic factor of Arrhenius—*R*·ln(*A*_ct_/Ω cm^2^)/(J K^−1^ mol^−1^) for the three acids at atmospheric pressure and separately. (●): HCl, (○): H_2_SO_4_, (▲): H_3_PO_4_, Solid line: fit in linear regression for both acids.

By similarity to the observed mutual correlation between the two Arrhenius parameters (*Ea* and ln*A*_ct_)^22^, likewise we have thought about inspecting the mutual dependence between the two current Arrhenius *E*a(*T*) and ln*A*_ct_(*T*) by plotting one parameter against the second for the three studied polyacids H_*x*_B. In fact, the Fig. 4 shows interesting causal correlation whether for each acid separately or for all three together, for which the quasi-linearity inter-dependence can be expressed as follows:

23 $$Ea\left(T\right)={E}_{a0}-{R\tau }_{0}\cdot {\text{ln}}{A}_{ct}(T)$$

where *E*_a0_ (Table 5) is a current activation energy corresponding the zero value of the entropic factor, and the slope *τ*_0_ (Fig. 4) is equivalent to an absolute temperature characteristic of the studied system under such conditions and within the working temperature range.

**Table 5:** Optimal parameters (*E*_a0_ and *τ*_0_), from linear regression of Eq. 23.

Acid	*E*_a0_	*τ*_0_	*τ*_0_	*R*-square
	kJ mol^−1^	K	°C	–
Both acids*	21.64	304.45	31.30	0.99971
HCl	18.952	320.48	47.33	0.99929
H_2_SO_4_	16.810	320.48	47.33	0.99931
H_3_PO_4_	17.082	320.15	47.00	0.99829
Both acids**	19.356	311.61	38.46	0.99898

*Fit in linear regression of (Eq. 23) for classical Arrhenius parameters as independent of temperature^1^. **Fit in nonlinear regression of (Eq. 23) for current Arrhenius parameters dependent on temperature.

Hence, some experimenters^1^ interpret the sign of the deviation to the Arrhenius linearity as a sub-Arrhenius or super-Arrhenius behaviors. Therefore, we notice that the activation energy *Ea* (Eqs. 15 and 17) can be interpreted as a potential energy barrier which is assumed to be dependent on temperature *Ea*(*T*).

In case of positive values of the activation energy derivative with respect to the reciprocal of absolute temperature at constant pressure: ${\left(\frac{\partial {E}_{a}(T)}{\partial (1/T)}\right)}_{P}$, super-Arrhenius behavior is observed, whereas for negative values, it is the sub-Arrhenius behavior. For this derivation, the values neighboring zero lead to the classical Arrhenius behavior (Eq. 17) and the *a*_1_-value tends to the classical Arrhenius activation energy *Ea*, independent of temperature (Table 4).

24 $${\left(\frac{\partial {E}_{a}(T)}{\partial (1/T)}\right)}_{P}=R{\left(\frac{{\partial }^{2}({\text{ln}}{R}_{ct})}{\partial {(1/T)}^{2}}\right)}_{P}={2a}_{2}+{6a}_{3}\left(\frac{1}{T}\right)+{12a}_{4}{\left(\frac{1}{T}\right)}^{2}+\dots +n(n{-1)a}_{n}{\left(\frac{1}{T}\right)}^{n-2}$$

Or else we can write the following:

25 $${\left(\frac{\partial {E}_{a}(T)}{\partial (1/T)}\right)}_{P}=-{T}^{2}{\left(\frac{\partial {E}_{a}(T)}{\partial (T)}\right)}_{P}$$

In our case, the system exhibits a super-Arrhenius behavior, *i*.*e*. the potential energy barrier is reduced whenever the temperature rises.

### Vogel–Fulcher–Tammann model

Generally, in case of clear deviation to the Arrhenius behavior, experimenters try to classify their results in the super-Arrhenius behavior or the sub-Arrhenius one^22^ or apply the Vogel–Fulcher–Tammann-type equation (VTF or VFT)^14,23–28^ especially when there is some divergence of experimental values at low temperature.

**Figure 5:**
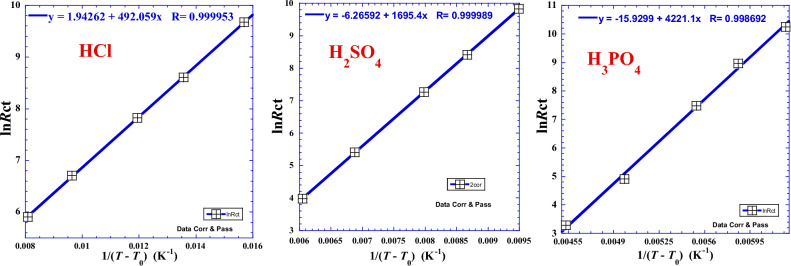
Variation of the logarithm of charge transfer resistance (ln*R*_ct_) against 1/(*T*–*T*_0_) for different acids.

**Figure 6:**
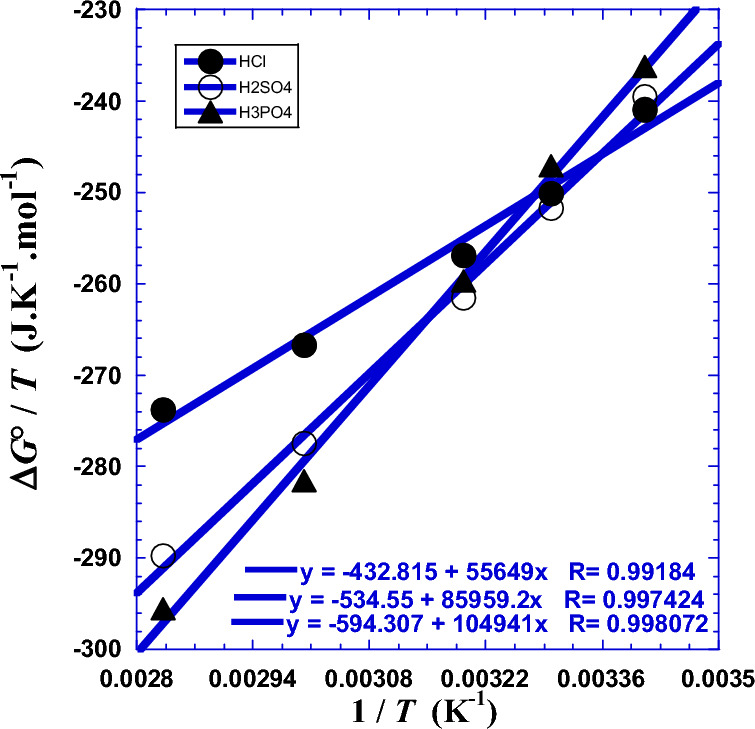
Variation of ΔG*/*T* against the inverse of the absolute temperature (1/*T*) for glassy Ni_82.3_Cr_7_Fe_3_Si_4.5_B_3.2_ alloy corrosion determined from impedance measurements in previous work^1^ for the three polyacids HCl, H_2_SO_4_ and H_3_PO_4_ at atmospheric pressure. (●): HCl, (○): H_2_SO_4_, (▲): H_3_PO_4_. Solid line: linear regression.

However, the variation of the logarithm of charge transfer resistance (ln*R*_ct_) against the inverse of the reciprocal temperature (1/*T*) exhibits a feeble deviation from the linearity of the Arrhenius behavior in our previous work (Fig. 1 of^22^) and in some results in literature^26,32–34^. In addition, regarding the suggested pseudo-hyperbolic behavior in previous work^22^ where the divergence of the variation of charge transfer resistance (*R*_ct_) for low temperatures (Fig. 6 of^22^) exhibits a kind of vertical asymptote, we propose to explore the Vogel–Fulcher–Tammann-type equation (VTF) which is characterized by a vertical asymptote and is used when the test of Arrhenius behavior fails^14,23–28^. In the case of the nonlinear behavior, it is found that the temperature dependence of charge transfer resistance can be physically fitted with the frequently VTF-type equation^14,23–28^ expressed as follows:

26 $$\mathit{ln}{R}_{ct}=\mathit{ln}{A}_{0}+\frac{{B}_{0}}{T-{T}_{0}}$$

where *A*_0_ and *B*_0_ are optimal constants and *T*_0_ is the Vogel temperature. It’s also interesting to use the modified VTF equation which is expressed as follow:

27 $$\mathit{ln}{R}_{ct}=\mathit{ln}{A}_{0}+\frac{{E}_{0}}{R}(\frac{1}{T-{T}_{0}})$$

where *R* is the perfect gas constant, *E*_0_ is the VTF activation energy and, *A*_0_ and, *T*_0_, are the pre-exponential factor and the Vogel temperature generally comparable to the glass transition temperature in viscosity property^14,23–28^. Experimental data are presented in Table 6 and presented in Fig. 5. For numerical applications, we can inject in Eq. 27, for each acid, the values of (*T*_0_), (ln*A*_0_) and (*E*_0_) from Table 6.

**Table 6:** Optimal coefficients (*A*_0_), Vogel temperature (*T*_0_),the logarithm of pre-exponential factor (ln*A*_0_), VTF activation energy (*E*_0_), *R*-square (*R*^2^) and standard deviation (*σ*) for the logarithm of charge transfer resistance (ln*R*_ct_) versus absolute temperature in VTF model (Eqs. 26 and 27) for different acids.

Acid	Values of parameters						*R*-square	SD
	ln*A*_0_	*B*_0_	*T*_0_	*T*_0_	*E*_0_	*A*_0_	*R*^2^	*σ*
	–	K	K	°C	kJ mol^−1^	Ω cm^2^	–	
HCl	1.294262	492.059	229.45	− 43.70	4.0912	3.6483	0.999906	0.00034
H_2_SO_4_	− 6.26592	1695.4	187.80	− 85.35	14.096	0.00190	0.999978	0.00065
H_3_PO_4_	− 15.9299	4221.1	132.50	− 140.65	35.096	1.2071 × 10^–7^	0.997386	0.00097

The Eq. 27 shows that when the temperature (*T*) tends toward (*T*_0_), it implies that the charge transfer resistance (*R*_ct_) becomes infinity, which indicates that the corrosion is inhibited. We see that (*T*_0_) decreases whenever the proticity (*x*_H_) of the acid increases (Table 6) while the VTF-energy (*E*_0_), which is in close relation with the activation energy (*Ea*), varies in the reverse sense.

### Correspondence between the two models

Generally, several experimenters manipulate physical and chemical quantities using various models for comparison and to develop discussions, interpretations and conclusions. This section falls within the scope of the correspondence between the modified Arrhenius equation (Eq. 3) and the VTF model (Eq. 27) to optimize the number of the models commonly utilized for investigations.

When we inject the second member of the VTF expression (Eq. 27) in Eqs. 15 and 16 which are deduced from the principal modified Arrhenius equation (Eq. 2) we can find the new expressions (Eqs. 14 and 15) of the Arrhenius parameters (*E*a) and (ln*A*_ct_) by comparing term by term without the direct use of the classical Arrhenius-type equation (Eq. 2).

28 $${E}_{a}(T)=\frac{{T}^{2}}{{(T-{T}_{0})}^{2}}\cdot {E}_{0}$$

29 $${\text{ln}}{A}_{ct}\left(T\right)= {\text{ln}}{A}_{0}-\frac{{T}_{0}}{{R(T-{T}_{0})}^{2}}\cdot {E}_{0}$$

Analyzing the expression of (Eqs. 14 and 15) we can conclude that the two parameters (*E*_0_) and (ln*A*_0_) of the VTF model are simply mathematical intermediary of calculation, except the third one (*T*_0_) which has physical significance indicating that the charge transfer resistance value (*R*_ct_) diverges and reaches a very high value when the temperature (*T*) is very close to (*T*_0_). Moreover, we can say that the non-zero (*T*_0_)-value is the principal cause of the deviation to the linearity of Arrhenius behavior. In fact, we can see in (Eqs. 14 and 15) that when (*T*_0_) tends to zero, the two parameters (*E*_0_) and (ln*A*_0_) become identical to those of Arrhenius (*Ea* and ln*A*_ct_) and in only this situation the two VTF parameters (*E*_0_) and (ln*A*_0_) have a full physical meaning. Furthermore, some theorists state that the Vogel temperature (*T*_0_) is in causal correlation with the corresponding glass transition temperature (*T*_*g*_)^14,23–28^.

## Thermodynamic parameters-temperature dependence

Treatments of the free Gibbs energy-temperature dependence considering the corresponding thermodynamic parameters (Δ*H°*) and (Δ*S°*) are rarely done in literature^32–34^. The majority consider approximately the constancy of theses parameters and interpret globally the phenomenon governing their studied systems based on their signs (positive or negative), their amounts (high or low values) and not on the eventual slow variation with temperature.

### Case of constant thermodynamic parameters

An alternative form of Arrhenius equation is the transition state equation^1,35,38–44^:

30 $${R}_{ct}=\frac{RT}{{N}_{A}h}{{\text{e}}}^{(\frac{\Delta H^\circ }{RT}-\frac{\Delta S^\circ }{R})}$$

where (*h*/J s): is Plank constant, (*N*_*A*_/mol^−1^): Avogadro's number, (*T*/K): absolute temperature, (*R*_ct_/Ω m^2^): charge transfer resistance, (*R*/J K^−1^ mol^−1^): Perfect gas constant (Δ*S°*/J K^−1^ mol^−1^): the entropy of activation and (Δ*H°*/J mol^−1^): the enthalpy of activation. Most of experimenters use the following practical expression:

31 $${\text{log}}\frac{{R}_{ct}}{T}={\text{log}}\frac{R}{{N}_{A}h}-\frac{\Delta S^\circ }{2.303R}+\frac{\Delta H^\circ }{2.303R}(\frac{1}{T})$$

where the plot of log(*R*_*ct*_/*T*) as a function of 1/*T* gives generally a reliable straight line, with a slope of + Δ*H°*/2.303*R* and an intercept to the ordinate of (log *R*/*N*_*A*_*h*–Δ*S°*/2.303*R*).

However, regarding that the Gibbs free energy of activation (Δ*G*°) is defined as follows:

32 $${\Delta G}_{T}^{^\circ }={\Delta H}^{^\circ }-T{\Delta S}^{^\circ }$$

Te determine the two thermodynamic parameters (Δ*H°*) and (Δ*S°*), we have to plot the ratio of Gibbs free energy by temperature (Δ*G*°/*T*) with the reciprocal of absolute temperature (1/*T*).

Then, regarding the Eq. 32, the Eq. 31 can be reformulated as follows:

33 $${\Delta G}_{T}^{0}=RTln\frac{{N}_{A}h{R}_{ct}}{RT}$$

So, the plot of *R*·ln(*N*_*A*_*hR*_*ct*_/*RT*) as a function of 1/*T* gives a quasi-straight line, with directly a slope of (Δ*H°*) and an intercept to the ordinate of (–Δ*S°*). Results are presented in Table 7 and depicted in Fig. 6. The negative values of the Gibbs free energy (Δ*G°*) indicate that the process of the intermediate complex in the transition state for the corrosion of Ni_82.3_Cr_7_Fe_3_Si_4.5_B_3.2_ alloy in acidic medium is spontaneous, while the positive values of enthalpy (Δ*H°*) show that the process is endothermic^1,22,30–37^.

**Table 7:** Results of values of the two thermodynamic parameters (Δ*H°*) and (Δ*S°*) obtained for linear regression by Eqs. 33 and 45 and nonlinear regression (Eqs. 44–49).

*T*	*T*	Δ*G*°	Linear Regression		Non Linear Regression	
			Δ*H°*	Δ*S°*	Δ*H°*	Δ*S°*
°C	K	kJ mol^−1^	kJ mol^−1^	J K^−1^ mol^−1^	kJ mol^−1^	J K^−1^mol^−1^
HCl						
20	293.15	− 70.626	55.649	432.815	80.850	516.72
30	303.15	− 75.822			71.176	484.90
40	313.15	− 80.440			62.120	455.25
47.55^a^	320.70^a^	− 83.766^c^			55.649^b^	432.815^b^
60	333.15	− 88.837			45.638	403.65
80	353.15	− 96.685			31.023	361.63
			*R*^2^ = 0.98279^d^		*σ* = 0.0277^e^	*σ* = 0.0213^e^
H_2_SO_4_						
20	293.15	− 70.238	85.959	534.55	108.00	608.02
30	303.15	− 76.294			99.542	580.03
40	313.15	− 81.890			91.620	554.08
47.55^a^	320.70^a^	− 86.007^c^			85.959^b^	534.55^b^
60	333.15	− 92.440			77.203	509.21
80	353.15	− 102.34			64.419	472.21
			*R*^2^ = 0.99458^d^		*σ* = 0.0120^e^	*σ* = 0.0265^e^
H_3_PO_4_						
20	293.15	− 69.240	104.941	594.307	119.61	644.21
30	303.15	− 74.907			113.98	623.08
40	313.15	− 81.320			108.71	606.82
47.55^a^	320.70^a^	− 86.037^c^			104.941^b^	594.307^b^
60	333.15	− 93.817			99.113	579.11
80	353.15	− 104.37			90.606	552.11
			*R*^2^ = 0.99786^d^		*σ* = 0.0057^e^	*σ* = 0.0287^e^

(a): Crossover temperature *T*_*cr*_ (Fig. 11a and b). (b); current thermodynamic parameters Δ*H°*(*T*) and Δ*S°*(*T*) calculated with Eqs. 48 and 49, respectively. (c): Gibbs free energy of activation (Δ*G°*) estimated with Eq. 44. (d): from^22^. (e): Standard deviation *σ.*

### Effect of protons’ number (***x***_H_)

**Figure 7:**
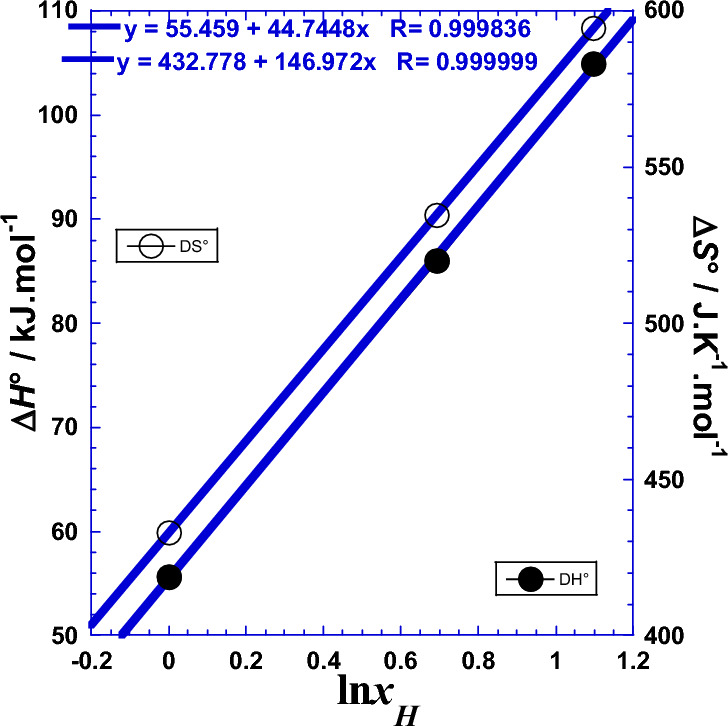
Variation against the logarithm of the acid proticity (ln*x*_H_) of (●): the enthalpy of activation (Δ*H°*/kJ mol^−1^) and (○): the entropy of activation (Δ*S°*/J K^−1^ mol^−1^).

Similarly with our previous work^1,22^ for the Arrhenius parameters, we will test the effect of the protons number (*x*_H_) of the polyacids (H_*x*_B). For that, we plotted the two thermodynamic parameters (Δ*H°*) and (Δ*S°*) against the logarithm of the acid proticity (ln*x*_H_). Figure 7 shows a linearity, which leads us to wonder if this relationship is valid for other strong acids with same proticity. The slope of the straight line in Fig. 7 represents the enthalpy gap *ε*_*g*_ (Table 8) and it corresponds to the jump of energy value when the proticity increases with unity. The same ascertainment is valid for the Fig. 7 concerning the entropy gap *σ*_*g*_ (Table 8). This observed linearity is similar to the Eqs. 8 and 9 with the same parameters values (Table 2) and it’s expressed as follows:

34 $${\Delta H}^{^\circ }({x}_{H})={{\Delta H}^{^\circ }}_{1}+{\varepsilon }_{g}\cdot {\text{ln}}{x}_{H}$$

35 $${\Delta S}^{^\circ }({x}_{H})={{\Delta S}^{^\circ }}_{1}+{\sigma }_{g}\cdot {\text{ln}}{x}_{H}$$

where the straight line parameters (Δ*H°*_1_) and (Δ*S°*_1_) correspond to the values of the enthalpy activation and entropy activation related to the monoacid HCl (*x*_H_ = 1), respectively (Table 8). Numerical applications require the use of the following convenient simple formulas:

**Table 8:** Straight lines parameters from linear regression of Eqs. 34 and 35.

*ε*_*g*_	Δ*H°*_1_	*σ*_*g*_	Δ*S°*_1_
kJ mol^−1^	kJ mol^−1^	J.K^−1^ mol^−1^	J K^−1^ mol^−1^
44.745	55.459	146.972	432.778
	55.649*		432.815*

*Correspond to HCl experimental data, see Table 6.

$${\Delta H}^{^\circ }/\mathrm{kJ }\;{{\text{mol}}}^{-1}=55.459+44.745\cdot {\text{ln}}{x}_{H}$$

$${\Delta S}^{^\circ }/\mathrm{J }{{\text{K}}}^{-1}\;{{\text{mol}}}^{-1}=432.778+146.972\cdot {\text{ln}}{x}_{H}$$

We expect that the values of these coefficients can vary according to the experimental conditions as well as the system characteristics and specificity.

However, Eqs. 30, 34 and 35 can be re-expressed to explicit the effect of number of protons (*x*_H_) of the polyacids (H_*x*_B) based on monoacid HCl (*x*_H_ = 1) data.

36 $${\Delta G}^{^\circ }({x}_{H})={{\Delta G}^{^\circ }}_{1}+{\gamma }_{g}\cdot {\text{ln}}{x}_{H}$$

37 $${\gamma }_{g}={\varepsilon }_{g}-T\cdot {\sigma }_{g}$$

38 $${{\Delta G}^{^\circ }}_{1}={{\Delta H}^{^\circ }}_{1}-T\cdot {{\Delta S}^{^\circ }}_{1}$$

where *γ*_*g*_ represents the free energy gap of acid protonation (*i.e*. when proticity has changed by one unit), Δ*H°*_1_ is the enthalpy of activation related to the monoacid HCl (*x*_H_ = 1) at given temperature and Δ*S°*_1_ is the entropy of activation. Numerical applications require the use of the following convenient simple formulas:

$${\gamma }_{g}/\mathrm{kJ }\;{{\text{mol}}}^{-1}=44.745-0.14697\cdot (T/{\text{K}})$$

$${{\Delta G}^{^\circ }}_{1}/\mathrm{kJ }\;{{\text{mol}}}^{-1}=55.459-0.43278\cdot (T/{\text{K}})$$

The values of these coefficients can vary according to experimental conditions as well as the system characteristics and specificity (Tables S1, S2 and S3).

We expect that these suggested expressions can be utilized in future estimations for other experimental conditions or other studied materials. We conclude that the proticity (*x*_H_) of the polyacid (H_*x*_B) has a substantial effect, which can be modeled for future prediction, or estimation and can induce theorists to develop or improve theories.

### Correlation between the Arrhenius parameters and thermodynamic ones

**Figure 8:**
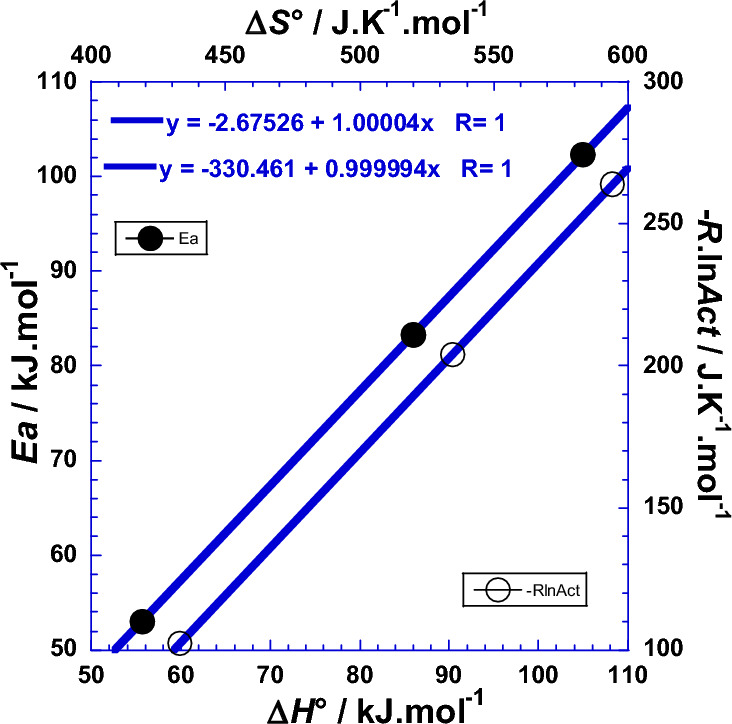
Correlation between the Arrhenius parameters and thermodynamic ones for glassy Ni_82.3_Cr_7_Fe_3_Si_4.5_B_3.2_ alloy. (●):correlation between the enthalpy of activation (Δ*H°*/kJ mol^−1^) and the activation energy (*Ea*/kJ mol^−1^); (○): correlation between the entropy of activation (Δ*S°*/J K^−1^ mol^−1^) and the entropic factor of Arrhenius—*R*·ln(*A*_ct_/Ω cm^2^)/(J K^−1^ mol^−1^).

Analysis of the enthalpy of activation (Δ*H*°)-values and those of the *Ea*, in the (Fig. 8), shows that the *Ea* and Δ*H*° values are very closely related. The same conclusion is also attributed to the correlation between the Arrhenius entropic factor (–*R*·ln*As*) and the entropy of activation (Δ*S*°). Starting from the fact that the values of the two slopes of (Fig. 8) are practically equal to unity (1.00004 and 0.999994), the following expressions are proposed.

39 $$\Delta H^\circ \, = Ea + {\updelta }H^\circ$$

40 $$\Delta S^\circ \, = {-}R\cdot{\text{ln}}A_{{{\text{ct}}}} + {\updelta }S^\circ$$

where (δ*H*° = 2.675 kJ mol^−1^) and (δ*S*° = 330.46 J K^−1^ mol^−1^) are the enthalpy increment and the entropy increment respectively. Numerical applications require the use of the following convenient simple formulae:

$$\begin{gathered} \Delta H^\circ /{\text{kJ}}\;{\text{mol}}^{ - 1} = Ea/{\text{kJ}}\;{\text{mol}}^{ - 1} + 2.675 \hfill \\ \Delta S^\circ /{\text{J}}\;{\text{K}}^{ - 1} \;{\text{mol}}^{ - 1} = {-}R\cdot{\text{ln}}(A_{{{\text{ct}}}} /\Omega {\text{ m}}^{2} )/{\text{J}}\;{\text{K}}^{ - 1} \;{\text{mol}}^{ - 1} + 330.46 \hfill \\ \end{gathered}$$

You have to be careful during discussions, interpretations and comparisons between amount values obtained for (*R*_ct_/Ω cm^2^) or (*R*_ct_/Ω m^2^), because the use of CGS or SI systems, which differ in the scale of base units, during the calculations of Arrhenius parameters or thermodynamic ones, doesn’t affect the values of the activation energy (*Ea*) and the activation enthalpy (Δ*H*°), while it gives difference of (± *R*·ln10^4^ =  ± 76.579 J K^−1^ mol^−1^) when calculating the Arrhenius entropic factor (–*R*·ln*A*_ct_) and the entropy of activation (Δ*S*°), and this is due to the conversion (cm^2^ ↔ m^2^).

We conclude that we can estimate one parameter when the other one is determined by any other technique. We notice that this shift is also due to the fact that the Arrhenius parameters represent the movement between two energy levels related to transition states, while the thermodynamic parameters, as state functions, represent the movement between two energy levels related to equilibrium thermodynamic states^1,22,30–37^. We add that the slopes values of the two straight lines of Fig. 8 are very near to the unity explaining then why that (*Ea* and Δ*H°*) and (– *R*·ln*A*_ct_ and Δ*S°*) have approximately the same value of gap or “jump” (*ε*_*g*_) and (*σ*_*g*_) when the number of protons (*x*) of the acid changes by one unity (Tables 2 and 8). Furthermore, mathematical comparison considering the expressions of Eqs. 1 and 2 and the Eqs. 30–33, which partly include both terms of Eqs. 1 and 2 in each member leads us to expect that the enthalpy increment (δ*H*°) is in close correlation with the contribution of thermal agitation on the activation enthalpy of the thermal stability related to the spontaneous formation of the hard non-reactive surface of passive film that inhibits the further corrosion (Tables S1, S2 and S3).

### Mutual correlation between the thermodynamic parameters

**Figure 9:**
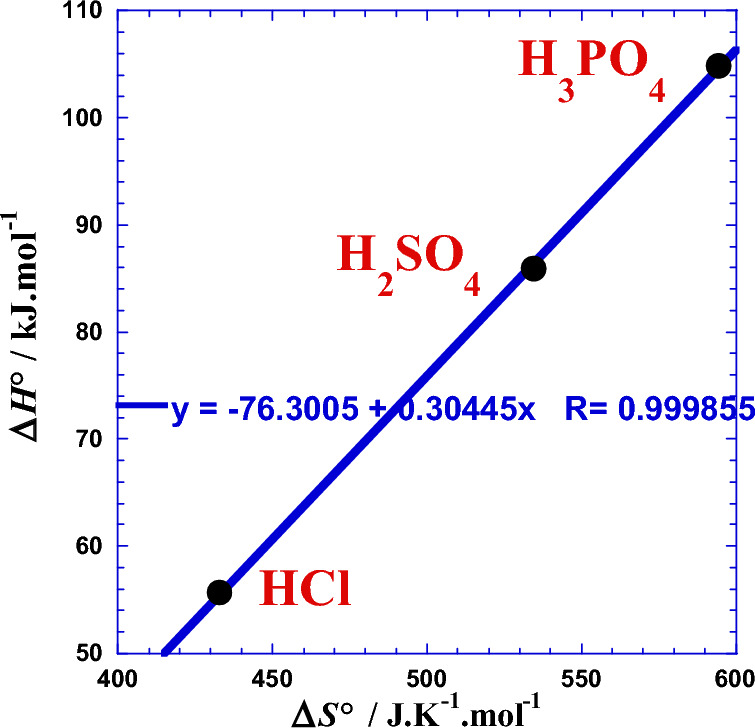
Mutual correlation between the enthalpy of activation (Δ*H°*/kJ mol^−1^) and the entropy of activation (Δ*S°*/J K^−1^ mol^−1^) for the three acids HCl, H_2_SO_4_ and H_3_PO_4_.

Analysis of the mutual correlation between the enthalpy of activation (Δ*H*°) and the entropy of activation (Δ*S*°) represented by the (Fig. 9) shows an excellent linearity which can permit us to estimate the free energy (Eq. 32) with only one thermodynamic parameter (Δ*H*°) or (Δ*S*°). This issue is interesting when we can theoretically predict one parameter; we can then deduce the value of the other one by this observed linearity (Tables S1, S2 and S3).

So, the linear enthalpy–entropy dependence observed in Fig. 9 can be expressed as follows,

41 $$\Delta H^\circ \left( T \right) \, = \tau_{0} \left[ {\Delta S^\circ \left( T \right) \, {-} \, \Delta S^\circ_{c} } \right]$$

where (*τ*_0_ = 304.45 K) and (∆*S*^o^_c_ = 250.618 J K^−1^ mol^−1^) are the characteristic temperature (Eq. 3) and the characteristic entropy of the studied system at such conditions, respectively. We can consider that the (*τ*_0_∆*S*^o^_c_)-product (Eq. 42) is equivalent to a characteristic enthalpy (∆*H*^o^_c_ = 76.301 kJ mol^−1^), the Eq. 41 can be rewritten as follows,

42 $$\Delta H^{o}_{c} = \tau_{0} \Delta S^\circ_{c}$$

43 $$\Delta H^{o} \left( T \right) \, = \tau_{0} \Delta S^{o} \left( T \right) \, {-} \, \Delta H^{o}_{c}$$

Numerical applications require the use of the following convenient simple formulas:

$$\begin{gathered} \Delta H^\circ \left( T \right)/{\text{J}}\;{\text{mol}}^{ - 1} = \, 304.45\left[ {\Delta S^\circ \left( T \right) \, {-} \, 250.618} \right] \hfill \\ \Delta H^{{\text{o}}} \left( T \right)/{\text{J}}\;{\text{mol}}^{ - 1} = \, 304.45\Delta S^{o} \left( T \right) \, {-} \, 76.301 \hfill \\ \end{gathered}$$

We deduce then, each investigated system has two main specific independent parameters (∆*H*^o^_*c*_) and (∆*S*^o^_c_), and a dependent parameter (*τ*_0_) which can be simply deduced by the Eq. 42. We note that (∆*S*°_*c*_) corresponds theoretically to the limit of the endothermicity, *i.e*. the activation entropy for which the activation enthalpy becomes zero and changes sign.

### Case of temperature-dependent thermodynamic parameters

Almost, all researchers fit thermodynamic behaviors of experimental data in linear regression to conclude about the global thermal character of the studied process (*i.e*. endothermic, etc.). Analyzing the abovementioned study of Arrhenius parameters-temperature dependence, we conclude that is better if we think about the fitting by nonlinear regression to reduce the discrepancy between the straight line and the uncertainty bars of some of experimental scatter points in order to obtain more accurate values of the two thermodynamic parameters (Δ*H°*) and (Δ*S°*). Then, the linearity of (Δ*G*°) with the absolute temperature (*T*) of Eq. 33 and that of (Δ*G*°/*T*) with the reciprocal of absolute temperature (1/*T*) are abandoned and replaced by a polynomial equation with two or three degrees expressed as follows,

44 $$\frac{{\Delta G}_{T}^{0}}{T}={a}_{0}+{a}_{1}\left(\frac{1}{T}\right)+{a}_{2}{\left(\frac{1}{T}\right)}^{2}+{a}_{3}{\left(\frac{1}{T}\right)}^{3}+\dots +{a}_{n}{\left(\frac{1}{T}\right)}^{n}$$

**Figure 10:**
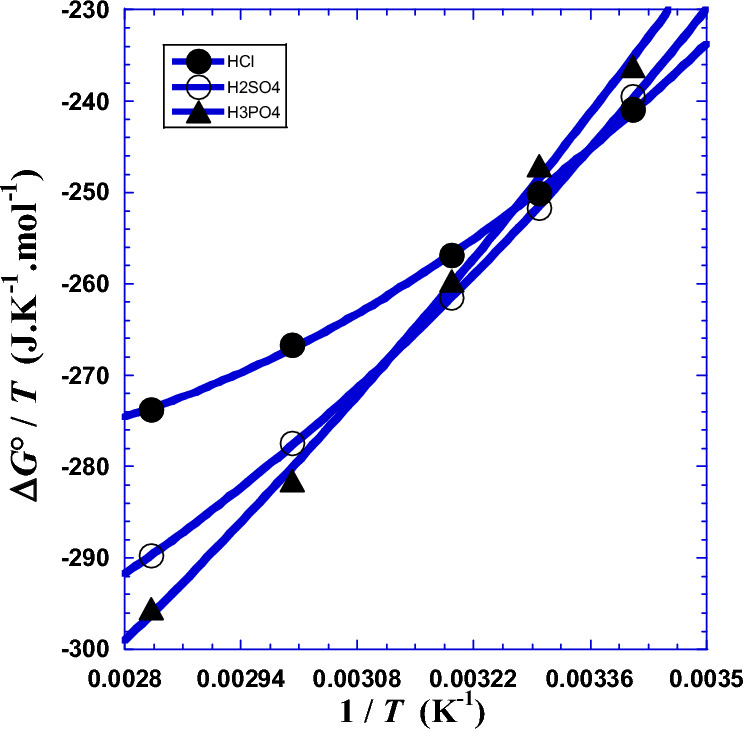
Variation of ΔG*/*T* against the inverse of the absolute temperature (1/*T*) for glassy Ni_82.3_Cr_7_Fe_3_Si_4.5_B_3.2_ alloy corrosion for the three polyacids HCl, H_2_SO_4_ and H_3_PO_4_. (●): HCl, (○): H_2_SO_4_, (▲): H_3_PO_4_. Curved lines: non linear regression (Eq. 44).

Given the deviation to the linearity of (Δ*G*°/*T*) with the reciprocal of absolute temperature (1/*T*) observed in literature^29–31^ is generally not very significant, we judge that we need to consider only a two-degree polynomial fitted in our nonlinear regression (Fig. 10) and acquire excellent approximation where the third coefficient is zero (*a*_3_ = 0)^1,22^.

The corresponding *a*_*i*_-coefficients of nonlinear fit are presented in the Table 9. We can clearly see that the statistical quality has improved quite well, and despite this we must pay attention to the fact that this could also be due to the small number of used working temperatures.

**Table 9:** Optimal *a*_*i*_-coefficients values obtained by non linear regression for Eq. 44 with second polynomial degree for glassy Ni_82.3_Cr_7_Fe_3_Si_4.5_B_3.2_ alloy corrosion for the three polyacids HCl, H_2_SO_4_ and H_3_PO_4_.

Acid	*a*_0_	*a*_1_	*a*_2_	*R*-square
	J K^−1^ mol^−1^	J mol^−1^	J K mol^−1^	–
HCl	− 16.768	− 212,423	4.29865e + 7	0.99935
H_2_SO_4_	− 170.621	− 148,531	3.76016e + 7	0.99992
H_3_PO_4_	− 352.116	− 51,109.9	2.50234e + 7	0.99766

The two main thermodynamic parameters, such as the enthalpy (Δ*H°*) and the entropy (Δ*S°*) can be determined from the basic thermodynamic Gibbs free energy relationship (Eq. 32).

Te determine values of the two thermodynamic parameters (Δ*H°*) and (Δ*S°*), we have to plot the ratio Gibbs free energy by temperature (Δ*G*°/*T*) with the reciprocal of absolute temperature (1/*T*). The mathematical handling of Eq. 32 can lead us to determine values of the two thermodynamic constant parameters (Δ*H°*) and (Δ*S°*) using the Eq. 45 in the case of linear behavior (*a*_2_ = 0 and *a*_3_ = 0 in Eq. 44) where the slope value of the straight line is (Δ*H*°) and intercept on the ordinate is (–Δ*S*°).

45 $$\frac{{\Delta G}_{T}^{0}}{T}={\Delta H}^{{\text{o}}}(\frac{1}{T})-{\Delta S}^{{\text{o}}}$$

Generally, the two thermodynamic parameters become variable with temperature and mathematical derivation of Eq. 32 can lead us to determine values of the two thermodynamic non-constant parameters Δ*H°*(*T*) and Δ*S°*(*T*) using the following equations.

46 $${\Delta H}^{{\text{o}}}(T) = {\left(\frac{\partial ({\Delta G}^{{\text{o}}}/T)}{\partial (1/T)}\right)}_{P}$$

47 $${\Delta S}^{{\text{o}}}\left(T\right)=\frac{{\Delta H}^{{\text{o}}}-{\Delta G}_{T}^{{\text{o}}}}{T}= -{\left(\frac{\partial ({\Delta G}^{{\text{o}}})}{\partial (T)}\right)}_{P}$$

Application of Eqs. 46 and 47 leads to the general following convenient general expressions.

48 $${\Delta H}^{o}\left(T\right)= {a}_{1}+{2a}_{2}\left(\frac{1}{T}\right)+3{a}_{3}{\left(\frac{1}{T}\right)}^{2}+ \dots +n{a}_{n}{\left(\frac{1}{T}\right)}^{n-1}$$

49 $${\Delta S}^{o}\left(T\right)= {-a}_{0}+{a}_{2}{\left(\frac{1}{T}\right)}^{2}+2{a}_{3}{\left(\frac{1}{T}\right)}^{3}+\dots +{(n-1)a}_{n}{\left(\frac{1}{T}\right)}^{n}$$

**Figure 11:**
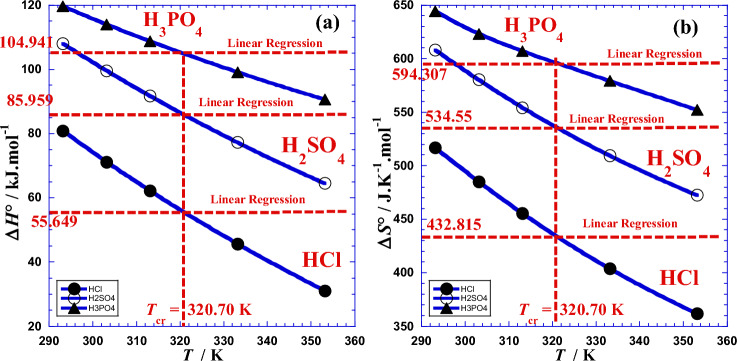
Variation against absolute temperature (*T*) of (**a**): the enthalpy of activation Δ*H*°(*T*) and (**b**): the entropy of activation Δ*S*°(*T*) for different acids. (●): HCl, (○): H_2_SO_4_, (▲): H_3_PO_4_.

Numerical applications require the use of the following convenient simple formulas:

$${\Delta H}^{o}\left(T\right)/k\mathrm{J }{{\text{mol}}}^{-1}={[a}_{1}+2{a}_{2}\left(\frac{1}{T}\right)]/1000$$

$${\Delta S}^{o}\left(T\right)/\mathrm{J }{{\text{K}}}^{-1} {{\text{mol}}}^{-1}={-a}_{0}+{a}_{2}{\left(\frac{1}{T}\right)}^{2}$$

where we can inject, for each acid, the values of (*a*_*i*_) from Table 9. The values of these coefficients can vary according to the experimental conditions as well as the system characteristics and specificity. Values of the two thermodynamic parameters (Δ*H°*) and (Δ*S°*) obtained by linear and nonlinear regressions are presented by Table 7 and depicted in Fig. 11 for the three acids.

Graphical analysis of (Fig. 11) shows that values of the two current thermodynamic parameters Δ*H*°(*T*) and Δ*S*°(*T*) meet those of classical thermodynamic parameters (Δ*H°*) and (Δ*S°*) (Table 7) for practically the same unique temperature (*T*_*cr*_ = 320.70K, 47.55 °C) named as crossover temperature which is approximately equal to the arithmetic mean (319.15 K, 46 °C) of the set of five working temperatures indicated in (Table 7). We can notice that this temperature can be an excellent working temperature for our studied system because it gives precise values of thermodynamic parameters whether we treat the data with linear or with non-linear regression.

We add that the crossover temperature (*T*_*cr*_) is not specific for the system like the characteristic temperature (*τ*_0_) studied in previous work^1^, one must be careful because it cannot be characteristic of the studied system; it is only an intermediate mathematical variable obeying Eq. 50 by simply indicating approximately the temperature of the system during its processing. The Eq. 50 can be obtained easily by equalizing Eq. 45 for classical parameters and Eq. 48 for current ones.

50 $${T}_{cr}= \frac{2{a}_{2}}{{\Delta H}^{{\text{o}}}-{a}_{1}}$$

where (Δ*H*°) represents the enthalpy of activation obtained by linear regression (Table 7).

For numerical applications, we can inject, for each acid, the values of (*a*_*i*_) from Table 9 and those of (Δ*H*°) obtained by linear regression from Table 7.

We notice that the middle-temperature (*T*_*md*_ = 323.15K;50 °C) of the studied temperature range is very close to the temperature parameter (*T*_*cr*_) as it is shown in Table 7. Indeed, to give an approximate estimation of the mean activation enthalpy Δ*H*° that should be obtained by classical linear regression we can apply the following reasoning. We can calculate the average value of the function Δ*H*°(*T*) expressed by Eq. 48 over a temperature domain [*T*_min_,*T*_max_] such as in our situation; [293.15,353.15]K, by the following expression:

51 $$\overline{{\Delta H}^{{\text{o}}}}=\frac{1}{{T}_{max}-{T}_{min}}{\int }_{{T}_{min}}^{{T}_{max}}{\Delta H}^{{\text{o}}}(T)dT$$

Which can be adapted, using Eq. 48, and can lead to a convenient expression (Eq. 52) for an average value of enthalpy of activation without using direct linear regression of (Δ*G*°/*T*) with 1/*T*.

52 $$\overline{{\Delta H}^{{\text{o}}}}=[{a}_{1}+2{a}_{2}\frac{ln\left(\frac{{T}_{max}}{{T}_{min}}\right)}{{T}_{max}-{T}_{min}}]$$

**Figure 12:**
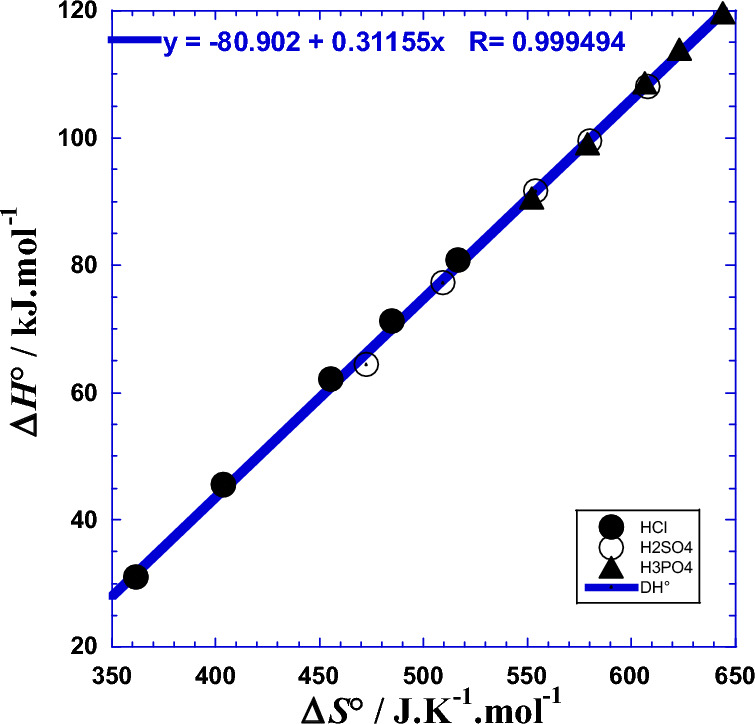
Mutual correlation between the enthalpy of activation (Δ*H°*/kJ mol^−1^) and the entropy of activation (Δ*S°*/J K^−1^ mol^−1^) for the three acids, both and separately. (●): HCl, (○): H_2_SO_4_, (▲): H_3_PO_4_, Solid line: fit in linear regression for both acids.

Inspired by to the observed mutual correlation between classical thermodynamic parameters (Δ*H°*) and (Δ*S°*) abovementioned, we similarly thought of inspecting their mutual dependence between the two current thermodynamic parameters. Δ*H*°(*T*) and Δ*S*°(*T*) by plotting one parameter against the second for the three studied polyacids H_*x*_B. In fact, the Fig. 12 shows interesting causal correlation, analogous to that of Eq. 43, whether for each acid separately or for all three together, for which the quasi-linearity inter-dependence can be expressed as follows:

53 $${\Delta H}^{{\text{o}}}\left(T\right)={\tau }_{0}\cdot {\Delta S}^{{\text{o}}}\left(T\right)-{{\Delta H}_{c}}^{{\text{o}}}$$

where ∆*H*^o^_c_ (Table 10) is a current activation enthalpy corresponding the null value of the activation entropy, and the slope *τ*_0_ (Fig. 12) is equivalent to an absolute temperature characteristic of the studied system at such conditions.

**Table 10:** Optimal thermodynamic parameters (∆*H*^o^_c_, ∆*S*^o^_c_ and *τ*_0_), from linear and nonlinear regression of Eqs. 41 and 43.

Acid	∆*H*^o^_c_	∆*S*^o^_c_	*τ*_0_	*τ*_0_	*R*-square
	kJ mol^−1^	J K^−1^ mol^−1^	K	°C	–
Both acids*	76.301	250.618	304.45	31.30	0.99971
HCl	84.269	262.947	320.48	47.33	0.99929
H_2_SO_4_	86.414	269.635	320.49	47.34	0.99931
H_3_PO_4_	86.028	268.711	320.15	47.00	0.99826
Both acids**	80.902	259.676	311.55	38.40	0.99899

*Fit in linear regression for thermodynamic parameters as independent of temperature.

**Fit in nonlinear regression for thermodynamic parameters dependent on temperature.

Another advantage, from which we benefit from the dependence on temperature, and other than that of the deduction of the endothermic character of the process, is that which indicates the sense of variation of the enthalpy with the temperature (Table 7) to know if this endothermicity is accentuated or attenuated and thus allowing us to choose the optimal working temperature. The same goes for the discussion and interpretation of entropy. In other words, the dependence with the temperature offers more explanations and fine interpretations than those deduced from only the constancy of thermodynamic parameters.

## Conclusion

In one previous work^1^, we have studied the effect of the temperature as well as the nature of polyacid medium (HCl, H_2_SO_4_ and H_3_PO_4_) on the tendency toward passivation and corrosion resistance for the nickel based glassy Ni_82.3_Cr_7_Fe_3_Si_4.5_B_3.2_ alloy.

In another previous work^22^, we have taken into account the deviation to the linearity of the Arrhenius behavior and modeled this particular dependence on temperature. Consequently, we have ended up at the following points. (*i*): The mutual correlation between the Arrhenius parameters, such as, the activation energy determined from polarization and impedance measurements and the entropic factor of Arrhenius permit to reveal a new concept of the current Arrhenius temperature. (*ii*): The causal correlation between the Arrhenius parameters and thermodynamic parameters, permit us to conclude that the activation energy can be considered, with a reliable approximation, as a thermodynamic function. (*iii*): The deviation to the Arrhenius linearity can be classified as a super-Arrhenius behavior for the studied system. (*iv*): The effect of the proton number in polyacid is quantified and modeled. (*v*): The novel suggested model describing a pseudo-hyperbolic behavior gives an excellent agreement (*R*-square ≈ 1) with the experimental data for each acid separately or for all three together.

As continuation of the abovementioned investigations, we keep on presenting furthermore original modeling of the temperature effect on the thermodynamic parameters as well as the effect of the polyacid proticity. Then, in the present work we extricate the following key points. (*i*): The mutual correlation between the Arrhenius parameters, such as, the activation energy determined from polarization and impedance measurements and the entropic factor of Arrhenius permit to rewrite the Arrhenius-type equation with only one parameter *Ea* or ln*A*_ct_ (Eqs. 5 and 6) and to facilitate, for theorists, to predict the value of one parameter (Eq. 3) when the other one can be predicted by certain theory or approximation. (*ii*): The effect of the proton number (*x*_H_) in polyacid (H_*x*_B) on the two Arrhenius parameters (*Ea* and ln*A*_ct_) is modeled by original expressions (Eqs. 8 and 9) for which we introduce the new concept of the activation energy gap *ε*_*g*_ and the entropic factor gap *σ*_*g*_ permitting to estimate new values of Arrhenius parameter *Ea* or ln*A*_ct_ of a polyacid, using a power law expression (Eqs. 10 and 11), when the same parameter of another acid is available (Table S2). (*iii*): The deviation to the Arrhenius linearity is simply modeled by a polynomial expression (Eq. 14) which reveals the new concept of current Arrhenius parameters depending on temperature (Eqs. 17 and 18) and then permits us to deepen the interpretation and discussion, and add additional elucidations of the effect of temperature on the Arrhenius parameters and the related phenomena governing the studied system. (*iv*): In the same context, we introduce the notion of crossover temperature (Eq. 19) as an optimal working temperature. (*v*): The mutual correlation between the current Arrhenius parameters, depending on temperature, exhibits the same behavior and interdependence whether for each acid separately or for all three together (Eq. 23). (*vi*): We introduced for the first time the Vogel–Fulcher–Tammann-type equation to model the variation of the charge transfer resistance with the respect of temperature (Eq. 27) which shows an excellent agreement with experimental data. (*vii*): In the same context, we have given expressions (Eqs. 28 and 29) permitting to link the VTF parameters and those of the Arrhenius-type equation to facilitate for the users to obtain double results by using only one chosen model. (*viii*): The effect of the proton number (*x*_H_) in polyacid (H_*x*_B) on the two thermodynamic parameters (Δ*H°* and Δ*S°*) is modeled by satisfying linear expressions (Eqs. 33 and 34) which permit us to estimate the parameters values to one polyacid, knowing those of the monoacid or another polyacid. (*ix*): The correlation between the Arrhenius parameters and the corresponding thermodynamic ones, such as (*Ea* and Δ*H*°) and (–*R*·ln*As* and Δ*S*°), leads us to determine the value of one parameter, simply by a gap (Eqs. 39 and 40) named enthalpy or entropy increment. (*x*): The mutual correlation between the thermodynamic parameters (Δ*H*° and Δ*S*°) shows a linear dependence (Eqs. 41–44) and reveals values of characteristic enthalpy and characteristic entropy, specific to the studied system in such conditions. (*xi*): The feeble deviation to the linearity of (Δ*G*°/*T*) *vs*. (1/*T*) permits us to express the dependence of the current thermodynamic parameters Δ*H*°(*T*) and Δ*S*°(*T*) on temperature (Eqs. 48 and 49) and to propose the notion of crossover temperature (Eq. 50) as an optimal working temperature. We conclude that even for the case of the current thermodynamic parameters depending on temperature, the interdependence maintains the linear behavior. (*xii*): we proposed predictive expression form for the charge transfer resistance–temperature dependence to approximately estimate the glass transition temperature (*T*_*g*_).

After all, it should be mentioned that the previous interpretations made based on the order–disorder or enthalpy–entropy compensation effect could be enhanced by raising the number of working temperatures. Likewise, it cans explain the tendency of these alloys to undergo a disorder-to-order transformation in certain temperature range. We note that some interfaces can introduce an order–disorder transition of this two-dimensional layered network into molecules, leading to increased diffusional characteristics and reduced bonding. Moreover, we can observe an order and disorder combined corrosion morphology of dual-phase Ni-based alloy in the passive state^22,32–37,45–49^.

Finally, we must be wary, an interpretation with few experimental data may lead to less certain or unconvincing conclusion.

### Supplementary Information

Supplementary Information.

## Data Availability

All data generated or analyzed during this study are included in this published article [and its supplementary information files].
