# Supplementary material for: Effect of acid proticity on the thermodynamic parameters of charge transfer resistance in corrosion and passivation of nickel based glass alloy
Source: Sci Rep. 2024 Jan 20;14:1815. doi: 10.1038/s41598-024-52036-0 (PMC10799951; doi:10.1038/s41598-024-52036-0)

**Supplementary Materials**

**Effect of acid proticity on the thermodynamic parameters of charge transfer resistance in corrosion and passivation of Nickel based glass alloy**

*Khadijah M. Emran*^1,*^, *Noureddine Ouerfelli*^2^.

^1^ Chemistry Department, Faculty of Science, Taibah University, Saudi Arabia.

^2^ Institut Supérieur des Technologies Médicales de Tunis, LR13SE07, Laboratoire de Biophysique et Technologies Médicales, Université de Tunis El Manar, Tunis, Tunisia.

* Corresponding author: Khadijah M. Emran E-mail: [kabdalsamad@taibahu.edu.sa](mailto:kabdalsamad@taibahu.edu.sa) (K.M. Emran).

Table S1 presents the thermodynamic properties and the Arrhenius temperature, and its derived properties as a function of the polyacids proticity (*x*).

**Table S1**. Arrhenius temperatures and thermodynamic parameters.


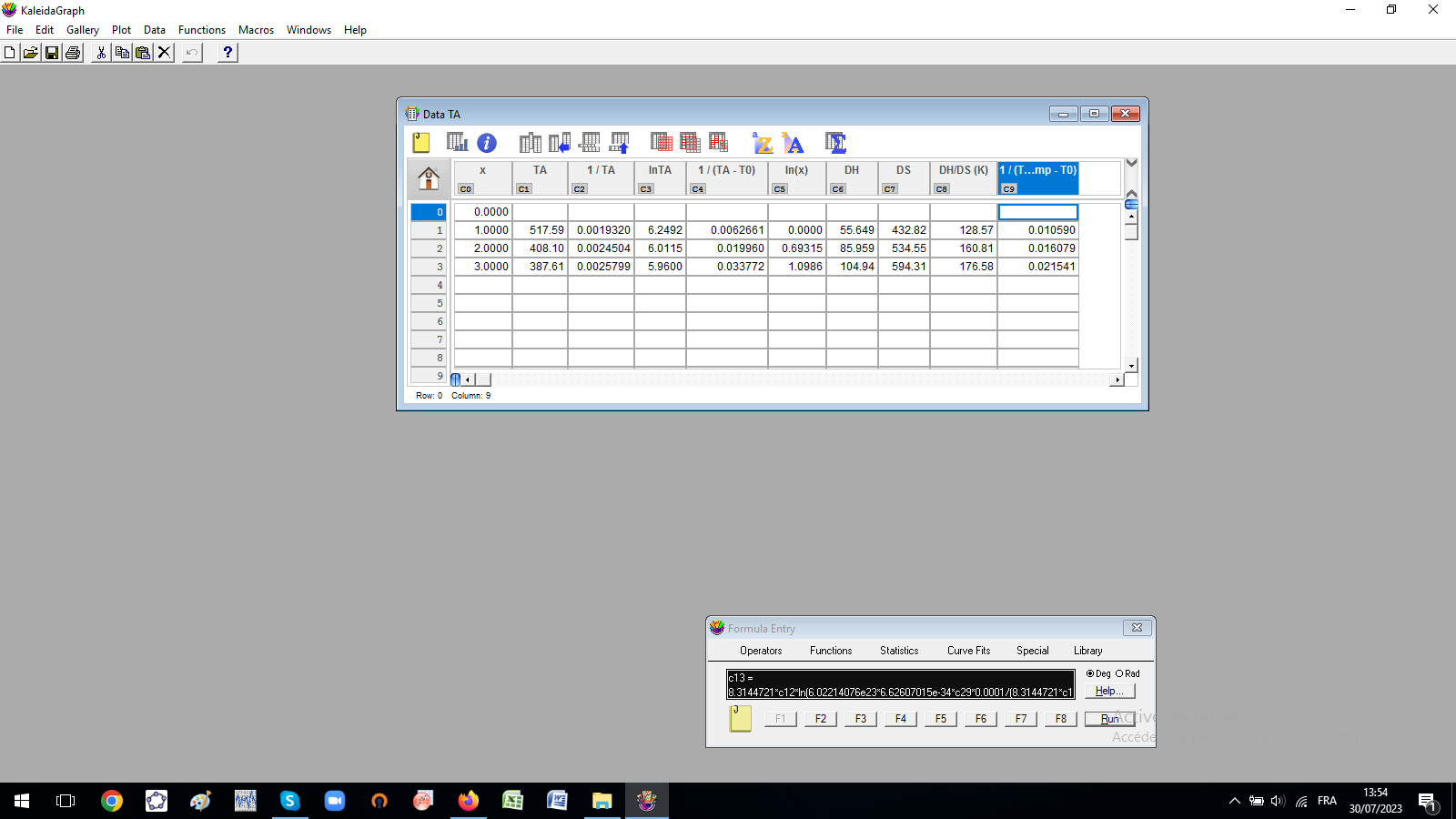


Table S2 presents the power law coefficients with relation to the thermodynamic properties and the Arrhenius temperature, and its derived properties as a function of the polyacids proticity (*x*) and their molecular weights (*M*).

**Table S2**. Arrhenius parameters and Power law coefficients.


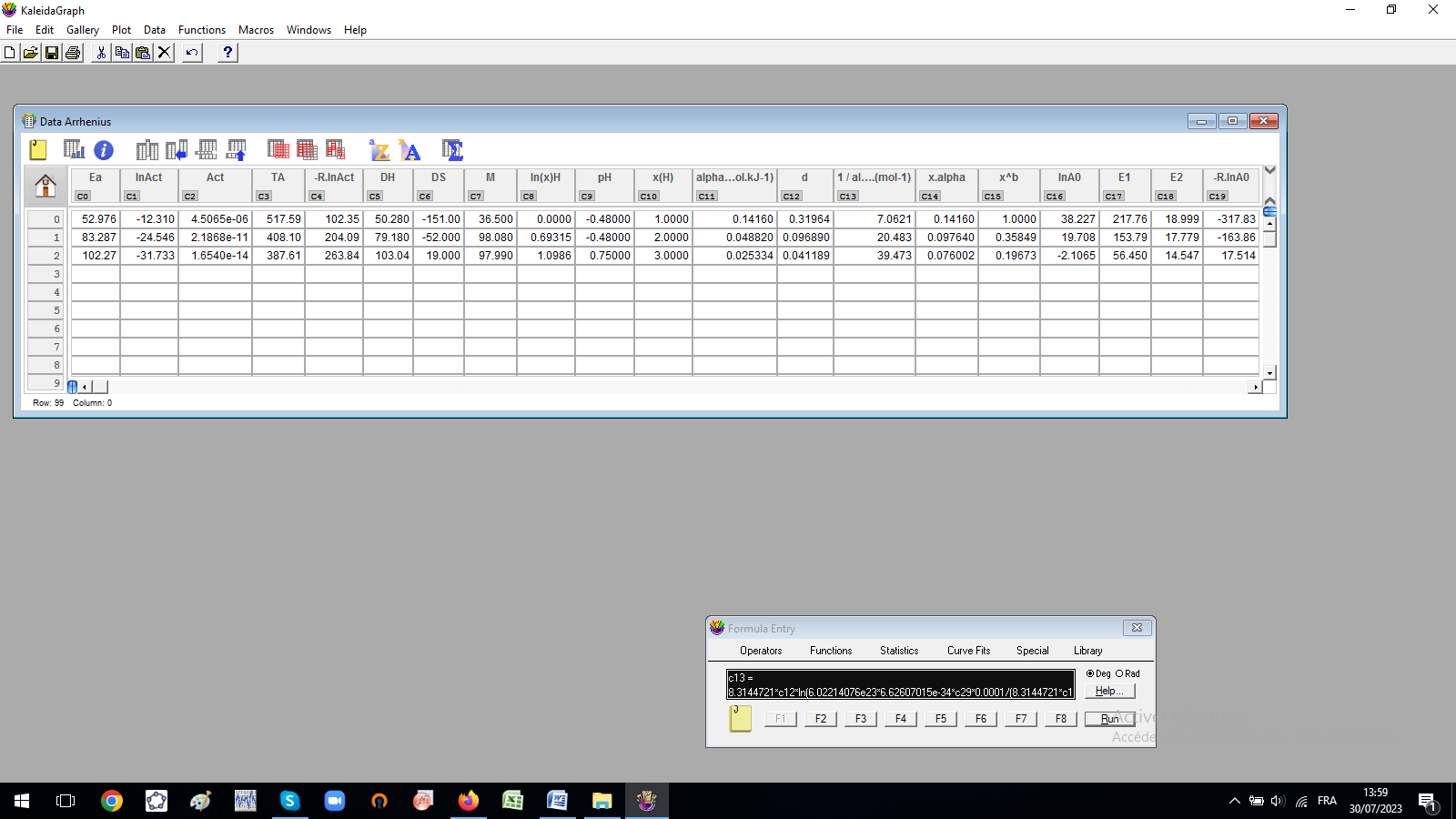


Table S3 presents the thermodynamic properties and Arrhenius parameters determined from polarization and impedance measurements.

**Table S3**. Thermodynamic parameters and correlation with protonicity.


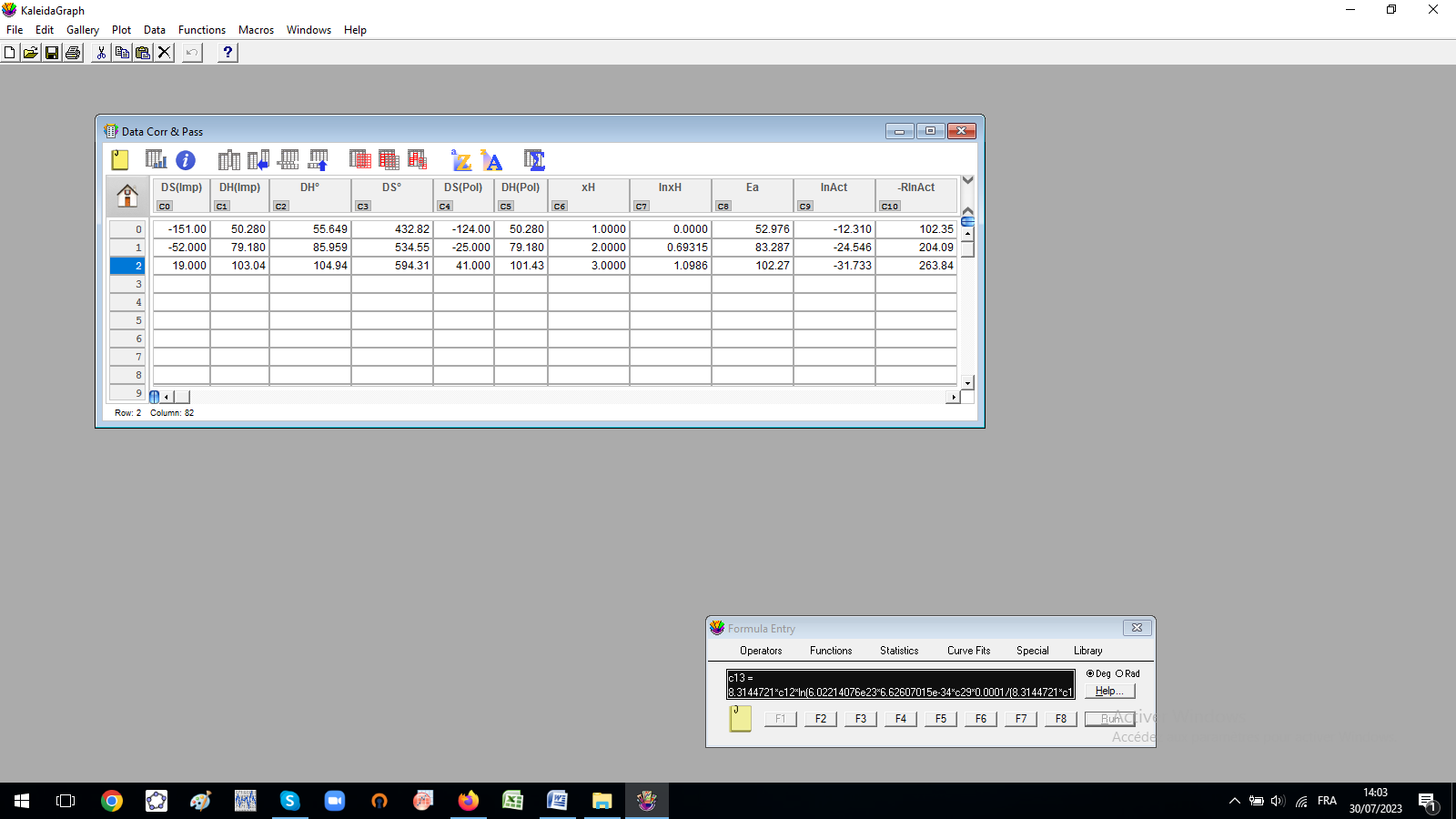

Supplement: Supplementary file 1 — Supplementary Information. [file 41598_2024_52036_MOESM1_ESM.docx]
